# Phenotypic and Transcriptomic Analyses of Autotetraploid and Diploid Mulberry (*Morus alba* L.)

**DOI:** 10.3390/ijms160922938

**Published:** 2015-09-22

**Authors:** Fanwei Dai, Zhenjiang Wang, Guoqing Luo, Cuiming Tang

**Affiliations:** Sericultural & Agri-Food Research Institute Guangdong Academy of Agricultural Sciences, 133 Yiheng Road, Dongguan Village, Tianhe District, Guangzhou 510610, Guangdong, China; E-Mails: daifanwei2011@163.com (F.D.); jiangwzh1982@126.com (Z.W.); guoqingluogd@163.com (G.L.)

**Keywords:** mulberry, autotetraploid, phenotype, transcriptome, plant hormone

## Abstract

Autopolyploid plants and their organs are often larger than their diploid counterparts, which makes them attractive to plant breeders. Mulberry (*Morus alba* L.) is an important commercial woody plant in many tropical and subtropical areas. In this study, we obtained a series of autotetraploid mulberry plants resulting from a colchicine treatment. To evaluate the effects of genome duplications in mulberry, we compared the phenotypes and transcriptomes of autotetraploid and diploid mulberry trees. In the autotetraploids, the height, breast-height diameter, leaf size, and fruit size were larger than those of diploids. Transcriptome data revealed that of 21,229 expressed genes only 609 (2.87%) were differentially expressed between diploids and autotetraploids. Among them, 30 genes were associated with the biosynthesis and signal transduction of plant hormones, including cytokinin, gibberellins, ethylene, and auxin. In addition, 41 differentially expressed genes were involved in photosynthesis. These results enhance our understanding of the variations that occur in mulberry autotetraploids and will benefit future breeding work.

## 1. Introduction

Mulberry (*Morus alba* L.) [1] is a woody plant native to China that is commercially valuable. The most important use of mulberry is as the sole food source of the domesticated silkworm (*Bombyx mori* L.). However, mulberry is also used in animal fodder, pharmaceuticals, food production, and landscaping [2,3]. Polyploidy is a heritable change in which the entire chromosome set is multiplied, and it plays an important role in plant evolution [4]. Two forms of polyploidy are often considered: allopolyploidy, which originates from interspecies hybrids, and autopolyploidy, which originates from intraspecies genome duplication events. Polyploidy is particularly widespread in the flowering plants (angiosperms), including many major crops [5]. Polyploid plants are often larger and have larger organs than their diploid relatives, including higher yield, larger leaves, larger fruit, more robustness, and some other agronomic characters [6,7,8], which makes polyploids quite appealing for agricultural breeding.

How are the larger plants regulated by the polyploidization? The most naïve hypothesis was that increase in gene copy number increased the amount of protein, which in turn increased the cell volume [9]. It was found that the ploidy-dependent increase in cell volume is genetically regulated in the experiment of investigating a wide range in cell size by tetraploidizing various mutants and transgenics of Arabidopsis thaliana [10]. Early research reported that polyploidization increased the chloroplast number and photosynthesis per cell, which may be due to increasing size of cells [11]. However, the mechanism behind the ploidy-related regulation of cell size, cell proliferation and expansion remains largely unclear. In recent years, plant breeders have worked with polyploids in mulberry and several artificially generated polyploids with “larger” mulberry characteristics have been reported [12,13,14]. Hence, we sought to investigate the physiological and molecular mechanisms for the enlargement phenomenon in mulberry polyploids.

Transcriptome-wide gene expression analysis has been demonstrated in many bred and natural polyploid plants. Research on transcriptional analyses of autotetraploids and their related diploids show a significant divergence in species-specific traits even though a great deal of common characteristics also exist [5,15]. For example, only ~1%–3% of genes are significantly differentially expressed between the autotetraploids and diploids of Arabidopsis (*Arabidopsis thaliana* L.), rice (*Olyza Sativa* L.), and Chinese woad (*Isatis indigotica* Fort.) [16,17,18], whereas ~10% of potato (*Solanum phureja* L.), birch (*Betula platyphylla* Suk.), and *Paulownia* (*Paulownia fortune* Hemsl.) genes are significantly differentially expressed [8,19,20]. In addition, RNA profiling using different tissues may partly cause transcriptome divergence. Among woody plants, the biosynthesis and signal transduction of indole-3-acetic acid and ethylene have been altered by a genome duplication event in birch [8], whereas differentially expressed transcripts are enriched in the energy metabolism pathway and in genetic information storage in *Paulownia* [20]. Hence, it is necessary to investigate the changing expression in key genes after polyploidization in the mulberry.

RNA-Seq is a powerful tool for detailed transcriptomic studies that is cost-efficient and yields a far greater amount of information than traditional sequencing technology [21]. In this study, we obtained a series of mulberry autotetraploids using a colchicine treatment and compared differences between the transcriptomes of diploid and autotetraploid mulberry plants using RNA-Seq technology. The results improved our understanding of the genetic regulation associated with mulberry autopolyploidization.

## 2. Results

### 2.1. Detection of Mulberry Autotetraploids

**Figure 1:**
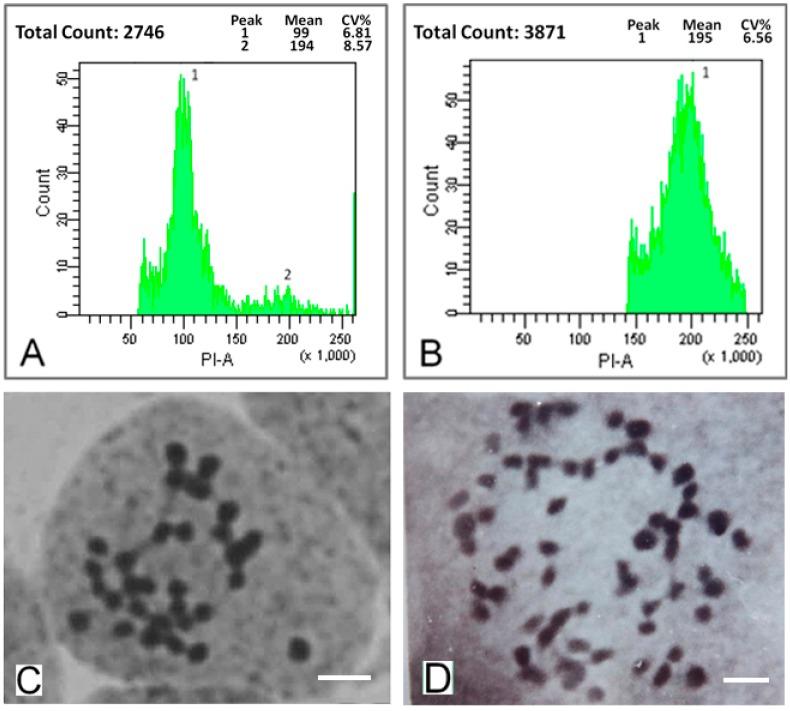
Ploidy analysis of mulberry leaves. (**A**) DNA content of diploids (the main peak is at channel 100); (**B**) DNA content of autotetraploids (the main peak is at channel 200); (**C**) Chromosome number in diploids (2*n* = 2*x* = 28). Bars = 5 μm; and (**D**) Chromosome number in autotetraploids (2*n* = 4*x* = 56). Bars = 5 μm.

A series of mulberry autotetraploids were generated with the colchicine treatment. We investigated the ploidy of two-month old mulberry seedlings using a flow cytometry analysis and chromosome counts. The DNA content of the control diploids had a main flow cytometry peak at channel 100 (Figure 1A). In contrast, the DNA content of the autotetraploids showed a main peak at channel 200 (Figure 1B). The chromosome count of diploids was 2*n* = 2*x* = 28, whereas that of the autotetraploids was 2*n* = 4*x* = 56 (Figure 1C,D). Of all 247 novel saplings generated with the colchicine treatment, about 49% saplings were autotetraploids while the rest of them were chimeras with part of polyploidization cell.

### 2.2. Phenotypic Changes versus Ploidy in Mulberry

**Figure 2:**
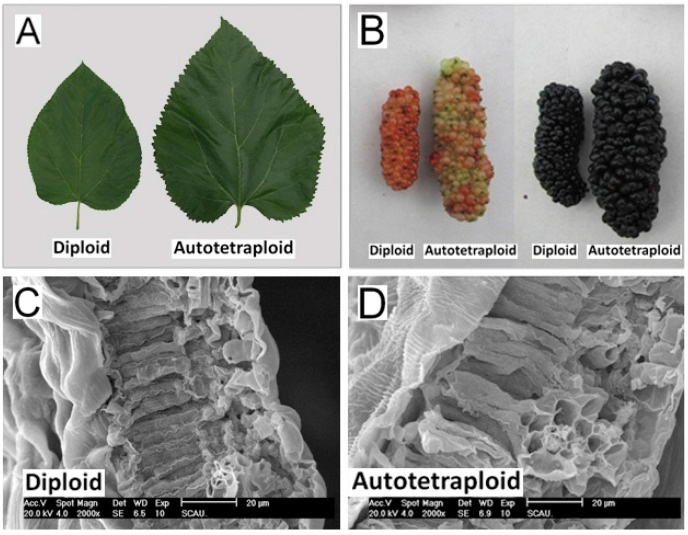
Phenotypic characterization of diploids and autotetraploids grown under the same conditions. (**A**) Leaves of diploids and autotetraploids; (**B**) Fruits of diploids and autotetraploids at the red (**left**) and black (**right**) fruit stages; (**C**) Leaf cross-section of a diploid; and (**D**) Leaf cross-section of an autotetraploid.

Diploid and autotetraploid mulberry trees at adult stage were characterized morphologically. The height, breast-height diameter, leaf area and cross-section, inflorescence length, fruit length and diameter of autotetraploid trees were larger than those of the diploid trees. The mean value of leaf area and leaf cross-section of autotetraploids were 99.28 cm^2^ and 2.88 μm, respectively, which were ~40% larger and ~19% thicker compared with those of diploids (Figure 2; Table 1). As shown in the leaf cross-section in Figure 2C,D, it appears that the cell size of spongy tissue and palisade tissue were larger in the autotetraploid leave, but the cell number did not reveal an apparent difference. Further, this was simply a direct observation, and with no statistical significance. The fruits of autotetraploids were larger than those of diploids at different fruit stages while the fruit maturation period had no significant differences. At the black fruit stage, the mean weight of autotetraploid fruit was 8.57 g, which was ~70% heavier than diploid fruit. And the mean value of fruit length and diameter were 5.70 and 2.22 mm, respectively, which were ~40% longer and ~55% greater compared with those of diploids (Figure 2; Table 1). The height and breast-height diameter of autotetraploid trees were also greater than those of diploids (Table 1).

**Table 1:** Phenotypic comparisons between diploids and autotetraploids.

Traits	Diploid	Autotetraploid
Height (m)	6.58 ± 1.35 A	7.81 ± 1.48 B
Breast-height diameter (cm)	8.34 ± 1.59 A	10.31 ± 1.72 B
Leaf area (cm^2^)	70.89 ± 4.52 A	99.28 ± 5.06 B
Fruit maturation period (day)	54.21 ± 1.38 A	53.47 ± 1.93 A
Fruit length (mm)	4.06 ± 0.18 A	5.70 ± 0.20 B
Fruit diameter (mm)	1.43 ± 0.11 A	2.22 ± 0.13 B
Fruit weight (g)	5.05 ± 0.29 A	8.57 ± 0.38 B
Leaf cross-section (μm)	2.42 ± 0.31 A	2.88 ± 0.30 B

Data are presented as mean ± standard deviation, *n* ≥ 10; Values labeled with different letters indicate a significant difference between autotetraploid and diploid plants at *p* < 0.05 levels, based on *t* test.

### 2.3. Gene Expression in Diploids and Autotetraploids

To compare differences between the transcriptomes of diploid and autotetraploid, cDNA libraries were generated from leaves of adult stage plants, and then Illumina paired-end sequencing was performed. A total of ~12 M, 50-bp, single-ended RNA-Seq reads were generated from each sample. There were three biological replicates from three separate trees each for diploid and autotetraploid. Each of the reads was mapped to the mulberry genome sequence, which contains 29,338 predicted genes [22]. All of the samples showed similar match results, with ~70% of reads matching the genome sequence and ~64% being unique matches. Additionally, ~62% of reads matched the predicted genes of the genome, with ~58% being unique matches. Approximately 20,000 predicted genes were covered, constituting ~68% of the total predicted genes of the genome (Table 2). The number of detected genes was saturated when the sequencing counts surpassed 5 million (Figure S1), and ~50% of the matched genes had >70% coverage by reads in all of the samples (Figure S2).

**Table 2:** The mapping of the sequencing data to the mulberry genome.

Samples		Total Reads	Total Base Pairs	Genome Mapping		Gene Mapping	
				Total Mapped Reads	Unique Match	Total Mapped Reads	Unique Match
Diploid	TL-1	12,436,222	609,374,878	8,519,806 (68.51%)	7,775,195 (62.52%)	7,641,432 (61.44%)	7,146,324 (57.46%)
	TL-2	11,715,021	574,036,029	8,164,792 (69.70%)	7,453,652 (63.62%)	7,326,122 (62.54%)	6,851,534 (58.49%)
	TL-3	12,017,012	588,833,588	8,381,379 (69.75%)	7,705,777 (64.12%)	7,544,926 (62.79%)	7,072,841 (58.86%)
Autotetraploid	YY56-1	11,838,555	580,089,195	8,340,481 (70.45%)	7,663,626 (64.73%)	7,478,695 (63.17%)	7,024,909 (59.34%)
	YY56-2	12,376,444	606,445,756	8,738,505 (70.61%)	7,965,117 (64.36%)	7,771,610 (62.79%)	7,287,705 (58.88%)
	YY56-3	11,784,921	577,461,129	8,272,019 (70.19%)	7,596,691 (64.46%)	7,468,606 (63.37%)	7,024,748 (59.61%)

### 2.4. Differential Gene Expression between Diploids and Autotetraploids

**Figure 3:**
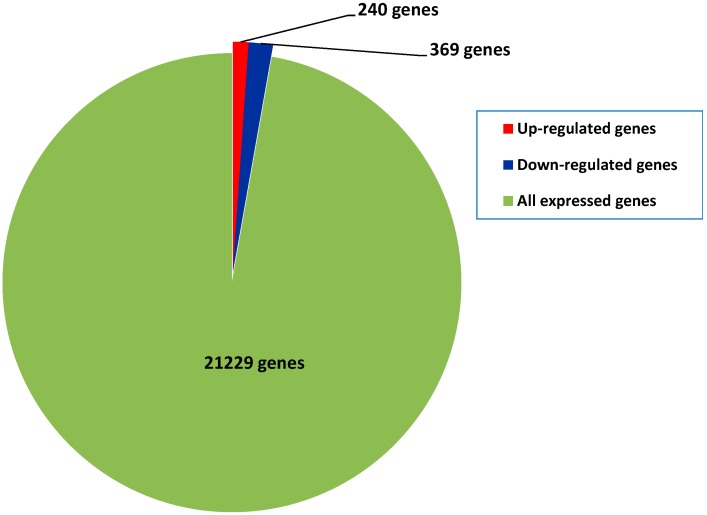
Number of differentially expressed genes identified between autotetraploids and diploids. The genes differentially up- and down-regulated in autotetraploids were determined comparing with corresponding samples of diploids (log_2_(RPKM_autotetraploid_/RPKM_di__ploid_) ≥ 1, and probability ≥ 0.8)

Among all of the samplings, 21,229 genes were detected. To compare the gene expression differences between diploids and autotetraploids, a reads per kilobase of exon per million reads mapped (RPKM) value for each sample was calculated. We considered genes with ≥2-fold changes in expression (log_2_(RPKM_autotetraploid_/RPKM_diploid_) ≥ 1) and probability ≥ 0.8 to be regulated genes. Based on this criterion, 609 (240 up-regulated and 369 down-regulated) differentially expressed genes were identified between diploids and autotetraploids (Figure 3; Tables S1 and S2). Among all of the expressed genes, only 2.87% of genes were differentially expressed. This indicated that gene expression was not highly altered after polyploidization in mulberry, which corroborates reports in other plant species [16,17].

**Figure 4:**
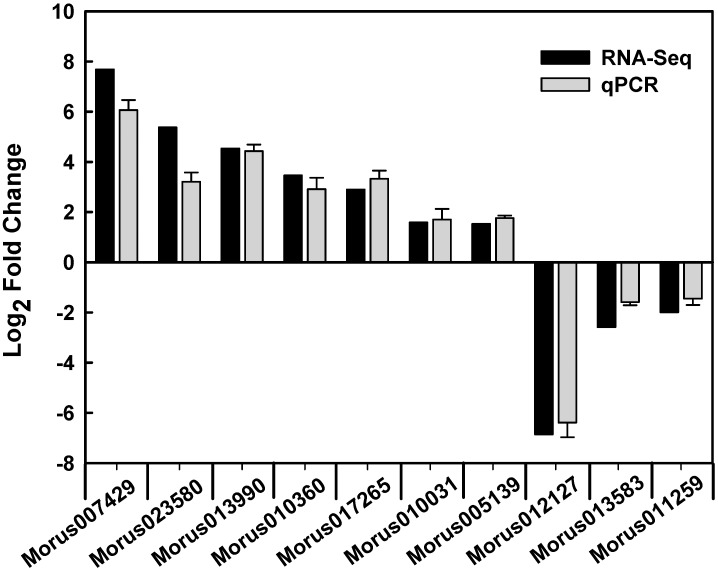
Verification of RNA-Seq results by qPCR.

To validate the gene expression data obtained through RNA-Seq, 10 genes were randomly selected from the differentially expressed genes for a quantitative real-time PCR (qPCR) analysis (Figure 4). The expression patterns of the 10 genes obtained through qPCR were largely consistent with the RNA-Seq data. The qPCR analysis confirmed that the RNA-Seq approach provides reliable differential gene expression data for ploidy analysis in mulberry.

### 2.5. Functional Classifications of Differentially Expressed Genes

**Figure 5:**
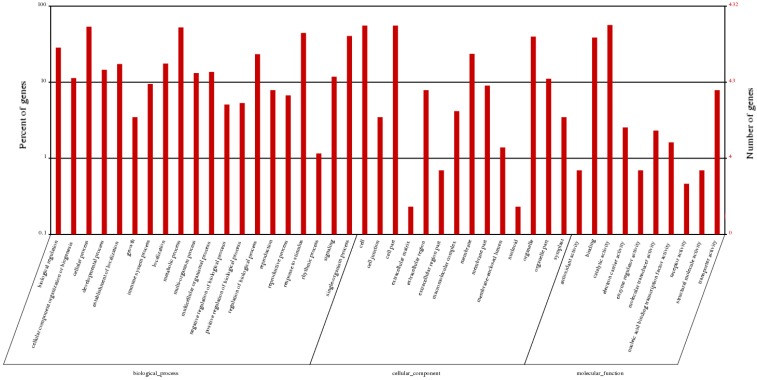
GO classifications of differentially expressed genes.

To further analyze the differentially expressed genes, we functionally classified these genes using the public gene ontology (GO) and Kyoto Encyclopedia of Genes and Genomes (KEGG) databases. Using the GO database, we characterized 432 differentially expressed genes were grouped into 44 GO terms based on sequence homology, which fell into three main groups—molecular function, biological process, and cellular component (Figure 5). For the biological processes, besides the large categories “cellular process” and “metabolic process”, genes were mostly enriched in “response to stimulus”, “single-organism process”, and “biological regulation”. In addition, the processes “localization”, “developmental process”, “multi-organism process”, and “signaling” differed significantly between autotetraploids and diploids.

**Figure 6:**
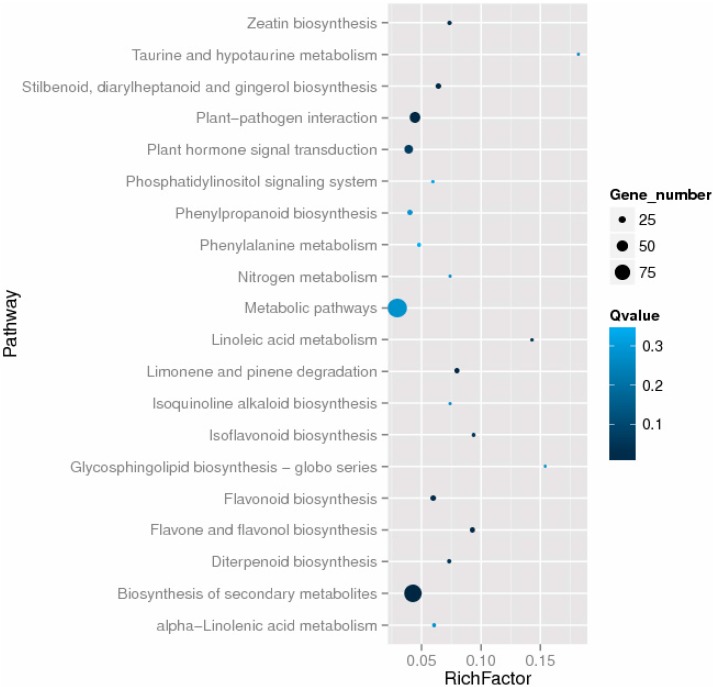
The top 20 enriched KEGG pathways among the differentially expressed genes.

Using the KEGG database, we annotated 350 differentially expressed genes to 100 KEGG pathways, using a blastx search with an *E*-value threshold of 1.0 × 10^−5^. The top 20 enriched KEGG pathways are shown in Figure 6. Besides the most highly represented group “metabolic pathways”, the “biosynthesis of secondary metabolites”, “plant–pathogen interaction”, and “plant hormone signal transduction” pathways were also highly enriched (Figure 6). Additionally, the pathways “flavone and flavonol biosynthesis”, “limonene and pinene degradation”, and “stilbenoid, diarylheptanoid and gingerol biosynthesis” differed significantly between autotetraploids and diploids.

### 2.6. Differentially Expressed Genes Related to Plant Hormones

Plant hormones are a category of important regulatory factors affecting plant growth and development. Among the differentially expressed genes between autotetraploids and diploids, a series of genes involved in the biosynthesis and signal transduction of plant hormones was found. Of the 609 regulated genes, 30 (4.9%) genes were associated with plant hormones (Table 3). Among them, six genes were related to cytokinin biosynthesis and signal transduction, including two adenylate isopentenyltransferases (Morus025042 and Morus010031), three two-component response regulator ARRs (Morus001125, Morus023955, and Morus023956), and one cytokinin dehydrogenase (Morus018596). The two adenylate isopentenyltransferase genes—important enzymes catalyzed during cytokinin biosynthesis [23]—were up-regulated in autotetraploids compared with the diploids of mulberry. Meanwhile, the three two-component response regulator ARR genes—which act as negative regulators of cytokinin signal transduction [24]—were down-regulated in the autotetraploids (Table 3).

There were five regulated genes associated with gibberellin (GA) signal transduction, including one GA receptor (Morus027556) and four DELLA proteins (Morus013990, Morus004260, Morus025266, and Morus025269). The GA receptor GID1—a hormone-sensitive lipase regulated by GA perception [25]—was up-regulated in autotetraploids compared with in diploids. DELLA proteins are key regulators of GA signaling, acting as negative regulators of GA responses [25]. Among the four regulated DELLA proteins in this study, three were down-regulated in the autotetraploids (Table 3). Nine regulated genes were involved in auxin biosynthesis and signal transduction, including auxin-induced protein, auxin-binding protein, auxin-repressed protein, and auxin hydrolase genes. Among the nine regulated genes, three were up-regulated whereas six were down-regulated in autotetraploids compared with diploids (Table 3).

Of the 10 regulated genes related to ethylene biosynthesis and signal transduction, two were 1-aminocyclopropane-1-carboxylate oxidase (ACO) genes (Morus004820 and Morus012808) and eight were ethylene response factor genes. ACOs are important enzymes during ethylene biosynthesis [26]. Several ethylene response factors play crucial roles during ethylene signal transduction [26]. In this study, among the 10 regulated genes related to ethylene, nine genes were down-regulated, whereas only one was up-regulated in autotetraploids compared with diploids (Table 3). These results suggested that plant hormones may play important roles in the phenotypic changes of autotetraploids compared with diploids.

**Table 3:** Differentially expressed genes involved in the biosynthesis and signal transduction of plant hormones. The fold change of each regulated gene is reported as log_2_, and the cutoff log_2_(RPKM_autotetraploid_/RPKM_diploid_) ≥ 1 and probability ≥ 0.8.

Accession Number	Annotation	log_2_(RPKM_autotetraploid_/RPKM_diploid_)	*p*-Value
**Cytokinin-Related Genes**			
Morus025042	Adenylate isopentenyltransferase	2.39	0.83
Morus010031	Cytokinin biosynthetic isopentenyltransferase	1.59	0.85
Morus018596	Cytokinin dehydrogenase	1.31	0.84
Morus001125	Two-component response regulator ARR9	−1.18	0.81
Morus023956	Two-component response regulator ARR9	−2.47	0.85
Morus023955	Two-component response regulator ARR9	−4.41	0.84
**GA-Related Genes**			
Morus027556	Gibberellin receptor GID1	8.47	0.99
Morus013990	DELLA protein GAI	4.54	0.91
Morus004260	GRAS1 protein (scarecrow-like protein 13)	−1.41	0.85
Morus025266	GRAS family transcription factor	−1.05	0.81
Morus025269	GRAS family transcription factor	−2.21	0.89
**Auxin-Related Genes**			
Morus016032	Auxin-induced protein 15A	2.63	0.81
Morus024575	Auxin-induced protein 22D	1.40	0.82
Morus015082	Auxin-repressed protein	1.06	0.83
Morus009595	Auxin-binding protein ABP19a	−1.21	0.84
Morus011104	Auxin-induced protein ARG2	−1.24	0.85
Morus010947	Auxin-induced protein 6B	−1.22	0.82
Morus012237	Auxin-induced protein 6B	−1.66	0.81
Morus019191	Indole-3-acetic acid-amino acid hydrolase	−1.25	0.82
Morus009587	Auxin-binding protein ABP19a	−7.62	0.97
**Ethylene-Related Genes**			
Morus010902	Ethylene-responsive transcription factor TINY	1.74	0.85
Morus011587	Ethylene-responsive transcription factor 4	−1.35	0.83
Morus018807	Ethylene-responsive transcription factor ERF	−1.44	0.81
Morus004820	1-Aminocyclopropane-1-carboxylate oxidase	−1.50	0.86
Morus012808	1-Aminocyclopropane-1-carboxylate oxidase	−1.53	0.86
Morus022972	Ethylene response factor 22	−1.80	0.87
Morus010314	Ethylene-responsive transcription factor 4	−2.09	0.89
Morus024017	Ethylene-responsive transcription factor 2	−3.47	0.92
Morus000238	Ethylene-responsive transcription factor 6	−3.67	0.93
Morus024526	Ethylene-responsive transcription factor 1B	−5.08	0.91

### 2.7. Differentially Expressed Genes Related to Photosynthesis

Photosynthesis is another important factor affecting plant growth. Among the regulated genes in the autotetraploids, 41 (6.9%) were involved in photosynthesis (Table 4). There were 14 genes expressed specifically in chloroplasts, including a phospholipase, thylakoid protein, aminotransferase, phosphate translocator, and polyphenol oxidase. Additionally, there were 20 cytochrome genes, including cytochrome b, cytochrome c, phytochrome interacting factor, and a series of cytochrome p450s. Seven other genes were associated with photosynthesis, including homogentisate phytyltransferase, photosystem I reaction center subunit XI, NADPH, and quinone oxidoreductase. Among these, the photosystem I reaction center subunit XI gene was up-regulated significantly in autotetraploids, with a log_2_(RPKM_autotetraploid_/RPKM_diploid_) value of 7.68. Another gene that encodes a homogentisate phytyltransferase—a key enzyme of the biosynthesis of the strong antioxidant tocopherol during photosynthesis [27]—was also up-regulated significantly in autotetraploids, with a log_2_(RPKM_autotetraploid_/RPKM_diploid_) value of 9.45.

**Table 4:** Differentially expressed genes involved in photosynthesis. The fold change of each regulated gene is reported as log_2_, and the cutoff log_2_(RPKM_autotetraploid_/RPKM_diploid_) ≥ 1 and probability ≥ 0.8.

Accession Number	Annotation	log_2_(RPKM_autotetraploid_/RPKM_diploid_)	*p*-Value
**Chloroplastic Genes**			
Morus016705	Transmembrane protein 45B, chloroplastic	4.27	0.89
Morus010360	Phospholipase A1-Igamma2, chloroplastic	3.46	0.86
Morus007099	Branched-chain-amino-acid aminotransferase 2, chloroplastic	2.87	0.85
Morus020529	Phosphoenolpyruvate/phosphate translocator 2, chloroplastic	2.02	0.82
Morus012122	Polyphenol oxidase, chloroplastic	2.03	0.87
Morus029233	Chloroplast latex aldolase	1.09	0.81
Morus017803	Chloroplastic lipocalin	−1.29	0.83
Morus022974	Allene oxide cyclase 3, chloroplastic	−1.14	0.82
Morus019289	Anthranilate synthase beta subunit 1, chloroplastic	−1.14	0.80
Morus016376	Pyruvate dehydrogenase complex, chloroplastic	−1.30	0.83
Morus009908	Omega-6 fatty acid desaturase, chloroplastic	−1.46	0.85
Morus021241	30S ribosomal protein S9, chloroplastic	−1.46	0.85
Morus024338	Thylakoid lumenal protein 2, chloroplastic	−1.55	0.84
Morus026216	Thylakoid membrane phosphoprotein, chloroplast precursor	−1.19	0.84
**Cytochrome Genes**			
Morus005139	Cytochrome C1	1.54	0.84
Morus008627	High chlorophyll fluorescence 153	1.02	0.80
Morus021180	Cytochrome P450	5.63	0.95
Morus021179	Cytochrome P450 71A1	4.78	0.94
Morus021181	Cytochrome P450 71A1	4.68	0.93
Morus015222	Cytochrome b5	−1.15	0.82
Morus013891	Phytochrome interacting factor 3	−1.21	0.83
Morus015343	Cytochrome P450 78A3	−1.08	0.82
Morus028259	Cytochrome P450 71D11	−1.10	0.82
Morus018959	Cytochrome P450 71A1	−1.20	0.83
Morus029652	Cytochrome P450	−1.58	0.85
Morus029651	Cytochrome P450	−2.22	0.85
Morus029646	Cytochrome P450 71D9	−2.25	0.82
Morus028261	Cytochrome P450 71D11	−2.76	0.83
Morus018592	Cytochrome P450 71D11	−3.78	0.90
Morus018954	Cytochrome P450 71A9	−4.05	0.84
Morus005453	Cytochrome P450 76C4	−4.17	0.90
Morus018591	Cytochrome P450	−4.33	0.90
Morus016212	Cytochrome P450 71D11	−4.60	0.81
Morus027395	Cytochrome P450	−6.38	0.93
**Other Genes**			
Morus007877	Homogentisate phytyltransferase 2	9.45	0.86
Morus007429	Photosystem I reaction center subunit XI	7.68	0.99
Morus009662	6-phosphogluconate dehydrogenase	2.09	0.88
Morus022319	Probable aldo-keto reductase 2	1.03	0.81
Morus022694	NAD(P)H-quinone oxidoreductase subunit N	−1.33	0.85
Morus012842	NADPH:cytochrome P450 reductase	−1.19	0.84
Morus001762	Quinone oxidoreductase 1	−1.15	0.81

## 3. Discussion

Polyploidy has played an important role in the evolution of angiosperms and was involved in the speciation of many important crops [28]. Autopolyploidy is usually associated with increased plant, organ, and cell sizes, so polyploids generated through plant breeding have been used as tools to increase crop yields [29,30,31]. By targeting relevant genes through transcriptomic techniques, the genetic basis of autopolyploidism has been investigated [19,20]. RNA-Seq is a newly developed high-throughput sequencing technology that provides a powerful and cost-efficient research platform for transcriptional profile analyses [32]. In this study, the transcriptomes of autopolyploid and diploid mulberry were investigated using Illumina RNA-Seq technology with mulberry genome sequences as the reference [22]. We obtained 609 transcripts that are differentially expressed between autopolyploids and diploids, accounting for ~2.87% of the total genome sequences.

Transcriptomic analyses have been performed in the generated autopolyploids of several plants, including Arabidopsis, potato, rice, Chinese woad, Rangpur lime (*Citrus limonia* Osbeck), birch, and *Paulownia* [8,16,17,18,33]. The percentage of differentially expressed genes in generated autopolyploids varies from 1.08% in Rangpur lime [33] to 12.6% in birch [8]. In herbaceous plants, leaflet and root tip tissues of potato has ~10% differentially expressed genes among different ploidies, whereas seedlings of Arabidopsis and pollen of rice have 1.63% and 2.59% between autotetraploid and diploid, respectively [16,17,19]. Compared to the other woody plants studied, mature leaves of mulberry had a higher percentage (2.87%) of differentially expressed genes than the 1.08% in mature leaves of Rangpur lime, but much lower than the 12.6% in shoot tips of birch or the 9.49% in young leaves from *Paulownia* [8,20,33]. Different tissues source for RNA profiling maybe another reason for transcriptome divergence. The percentages of differentially expressed genes between autopolyploids and diploids show species-specific and tissue-specific features.

Among the genes demonstrating expression changes between autopolyploids and diploids, plant hormone-related genes are an important category [8,16]. Ethylene- and auxin-related processes are controlled by highly regulated genes in autopolyploid Arabidopsis seedlings [16], and the biosynthesis and signal transduction of the auxin and ethylene pathways are altered after genome duplication in birch [8]. In Rangpur lime, GA- and auxin-related GO categories are over-expressed in autotetraploid plants [33]. In this study, among the 609 regulated genes in the autotetraploids, 30 (4.9%) genes were associated with plant hormones (Table 3). Cytokinin, GAs, and auxin—all of which are plant hormones that promote plant development and growth [34]—were significantly affected in autotetraploid mulberry compared with in diploid mulberry (Table 3).

There were six regulated genes related to cytokinin biosynthesis and signal transduction, and five regulated genes associated with GA signal transduction in the autotetraploids (Table 3). Among them, two adenylate isopentenyltransferase genes—important enzymes catalyzed [23] during cytokinin biosynthesis—were up-regulated, whereas three two-component response regulator ARR genes—which are negative regulators of cytokinin signal transduction [24]—were down-regulated in autotetraploid mulberry (Table 3). In the GA signal transduction pathway, a GA receptor, the GID1 gene, was up-regulated, whereas three genes encoding negative regulators of DELLA proteins [25] were down-regulated in the autotetraploids (Table 3). Levels of ethylene—a plant hormone suppressing development and growth [34]—were significantly altered in autotetraploid mulberry (Table 3). Of the 10 regulated genes related to ethylene, most of them were down-regulated in the autotetraploids (Table 3). In summary, plant hormones—especially cytokinin, gibberellin, and ethylene—may play important roles in the phenotypic changes of autotetraploids.

The rate of photosynthesis and chloroplast numbers both increase in association with ploidy increases [11,35,36]. In addition, photosynthesis-related genes are up-regulated in polyploidy plants [18,37]. In Arabidopsis, photosynthesis- and chlorophyll-related GO categories are enriched in autotetraploids compared with diploids [16]. In the present work, a series of differentially expressed genes that are involved in photosynthesis—including genes specifically expressed in chloroplasts, cytochrome genes, and photosystem-related genes—were up-regulated in autotetraploid mulberry (Table 4). Several important genes were substantially up-regulated in autotetraploids, such as the photosystem I reaction center subunit XI gene and the homogentisate phytyltransferase gene (Table 4). RNA for Illumina sequencing in this study were from mature leaves, the main tissues for photosynthesis, which may be another reason for so many different expressed genes related to photosynthesis. In brief, photosynthesis may be an important factor affecting phenotypic changes in autotetraploid plants.

The mechanism of polyploidization regulating larger organs has been studied for several years. Previous studies have reported that polyploidization increased the cell size, chloroplast number and photosynthesis per cell [10,11]. In this study, we investigated the leaf cross-section of diploid and autotetraploid mulberry. We seemed to observe the cell size increased in autotetraploid compared with diploid but the cell number did not reveal an apparent difference (Figure 2). On the transcriptome level, we found that gene expression of two important hormones, cytokinin and GAs—promoting plant growth and affecting cell size [34]—were positively regulated in autotetraploid mulberry (Table 3). Moreover, a series of photosynthesis related genes, including several chloroplast specifically expressed genes, were up-regulated in autotetraploid mulberry (Table 4). It could be speculated further that mulberry autotetraploid could increase level of cytokinin and GAs, which thereby increased the cell size and photosynthesis, ultimately resulted in larger organs. Further research on the mechanism of larger organs regulated by polyploidization is needed.

## 4. Experimental Section

### 4.1. Plant Materials

Seeds of diploid mulberry (*M. atropurpurea*) were soaked in 0.1% colchicine for 48 h in the dark to induce autotetraploidy, and seeds soaked in distilled water under the same conditions acted as controls. The seeds were sown in a greenhouse after colchicine treatment, and 247 novel saplings and 39 control diploid saplings were transplanted into plastic pots in a greenhouse.

### 4.2. Ploidy Measurement

The DNA content of the leaves of two-month old seedlings was evaluated by flow cytometry using the methods of Galbraith *et al.* [38] with some modifications. Three biological replicates performed of each sample. Briefly, ~0.5–1 cm^2^ young intact leaves were chopped in 1 mL of ice-cold extraction buffer (50 mM MgCl_2_, 50 mM citric acid, 5 mM HEPES, 0.1% Triton X-100, and 1% PVP-40) using a new razor blade. The crude suspension was filtered through a 42-μm nylon filter to remove cell debris and then added to a propidium iodide staining solution to a final concentration of 50 μg/mL. After 1 h of incubation at room temperature, the fluorescence intensity was measured using a FACSAriaII (BD Biosciences, San Jose, CA, USA) flow cytometer with excited blue light at 488 nm and 5 × 10^8^ J/s. The percentages of the cells that showed varied DNA contents were determined using BDFACSDiva software, and the DNA content of diploid leaves in the control group were measured in parallel.

Mitotic chromosomes were counted in young leaf bud of two-month old seedlings. Three biological replicates performed of each sample. Buds were collected and treated with a fixative buffer (concentrated hydrochloric acid/45% acetic acid/ethanol at 2:1:1 (*v*/*v*/*v*)) for 5 min. After flushing with water two or three times and immersing in water for 10 min, leaf buds were placed onto microscope slides and stained with one drop of carbolfuchsin and one drop of 45% acetic acid. Then, leaf buds were mashed with tweezers, the cytoplasmic residue was cleared, and the remains covered with glass. The chromosomes were visualized under a microscope (AxioScope A1, Carl Zeiss MicroImaging, Göttingen, Germany) using 1000× magnification. Approximately 10 metaphase cells were assessed for each leaf bud.

### 4.3. Phenotype Measurement

A series trees of autotetraploid (YY56) and diploid (TL) were adjacent planted in a plantation with the same parcel and climate condition. Ten adult stage (three-year old) mulberry trees of each cultivar were used in phenotype measurement. Leaf cross-sections were evaluated using scanning electron microscopy. An area ~1 cm^2^ from the center of mature leaves was fixed for 24 h in 2.5% glutaraldehyde fixation solution containing 2.5% glutaraldehyde and 0.1 M phosphate-buffered saline (PBS, pH 7.4) and dehydrated using a graded series of alcohol-isoamyl acetate concentrations, each for 15 min. The samples were dried using a critical point dryer (HCP-2, Hitachi, Tokyo, Japan), mounted on scanning electron microscopy stubs, sputter-coated with gold using an ion coater (Eiko IB-5, Hitachi), and observed under a scanning electron microscope (Philips XL30, Philips Electron Optics, FEI UK Ltd., Cambridge, UK). At least five leaf samples from each individual tree were viewed and measured at 20 kV using 2000× magnification. Leaf areas were measured from at least 10 healthy and fully expanded leaves collected at random from each tree. Height and breast-height diameters were measured from ten trees. The lengths, weights and diameters of the fruits were measured using a Vernier caliper, and the fruit maturation period were measured from end of the flowering to black fruit stage. At least 10 healthy fruits were selected at random from each tree and measured. SIGMASTAT from SPSS [39] was used to analyze the morphological data. Student’s *t* test were used to detect differences between autotetraploid and diploid at the usual probability level *p* = 0.05.

### 4.4. RNA Extraction, Illumina Sequencing, and Data Processing

For Illumina sequencing, three biological replicates from six independent adult stage trees of autotetraploid and diploid were used. Total RNA from each sample was extracted using the RNAiso Plus reagent (Takara BIO Inc., Otsu, Japan) and further purified using RNeasy Plant Mini kit reagents (Qiagen, Valencia, CA, USA). RNA quality was verified using a 2100 Bioanalyzer RNA Nanochip (Agilent, Santa Clara, CA, USA). All samples had an RNA integrity value of >7.5. RNA was then quantified using a NanoDrop ND-1000 Spectrophotometer (Nano-Drop, Wilmington, DE, USA). Total RNA (10 μg) was prepared for the cDNA library for each pool.

Illumina sequencing was performed at the Beijing Genomics Institute, Shenzhen, China using the HiSeq 2000 platform (Illumina, San Diego, CA, USA). First, poly-T oligo-attached magnetic beads (Illumina) were used to isolate poly(A) mRNA from total RNA. The purified mRNA was then fragmented into 200- to 700-nt pieces. The first strand of cDNA was synthesized using random hexamer primers, followed by synthesis of the second strand using SuperScript Double-Stranded cDNA Synthesis kit reagents (Invitrogen, Camarillo, CA, USA). The synthesized cDNA was subjected to end repair and phosphorylation using T4 DNA and Klenow DNA polymerases and T4 polynucleotide kinase, respectively. The repaired cDNA fragments were 3ʹ-adenylated using the Exo-Klenow fragment, and the Illumina paired-end adapters were ligated to the ends of these 3ʹ-adenylated cDNA fragments. To select templates for downstream enrichment, the products of the ligation reaction were purified by electrophoresis in a Tris-acetate-EDTA (2% *w*/*v*) agarose gel. cDNA fragments (200 ± 25 bp) were excised from the gel. Fifteen rounds of PCR were performed to enrich the purified cDNA templates using PCR primers PE 1.0 and PE 2.0 (Illumina) with Phusion DNA polymerase. Finally, after validation on an Agilent Technologies 2100 Bioanalyzer using Agilent DNA 1000 Chip kit reagents, the cDNA library was constructed by 50-bp, single-end RNA sequencing (RNA-Seq) in a PE flow cell using the Illumina Genome Analyzer HiSeq 2000.

Sequencing quality was evaluated and the data were summarized using the Illumina/Solexa pipeline software. Library saturation was also analyzed. For the raw data, adaptor sequences were eliminated, and distinct clean reads were identified. Subsequently, clean reads and distinct clean reads were classified based on their copy numbers within the library, and the percentages of total clean and distinct reads were calculated. The raw data have been deposited in the National Center for Biotechnology Information (NCBI) Gene Expression Omnibus (GEO) database [40] under submission number GSE70428.

### 4.5. Annotation and Analysis of Sequence Data

For annotation, all sequences were mapped to the mulberry genome [22]. The expression levels of each gene were estimated using the frequency of clean reads and then normalized to RPKM [41]. Differential expression genes analysis was used the NOIseq metheod [42]. Fold changes were assessed using the log_2_ ratio after expression abundances were normalized to RPKM. Differential expression genes were cutoff log_2_(RPKM_autotetraploid_/RPKM_diploid_) ≥ 1 and probability ≥ 0.8. Sequences were characterized using the GO database (http://www.geneontology.org/) [43]. Pathway assignments were determined with the KEGG pathway database [44] using the blastx algorithm with an *E*-value threshold of 1.0 × 10^−5^. The differential gene expression analysis data have been submitted to the GEO database under submission number GSE70428.

### 4.6. qPCR and Statistical Analysis

Mature leaves of adult stage mulberry trees were used in qPCR. Primers were designed using Primer 5.0 software [45]. Mulberry *MaACT3* (GenBank accession number: HQ163775) gene was used as the reference gene. Expression levels for all of the candidate genes were computed based on the stable expression level of the reference gene. qPCR was performed in 96-well plates on a Roche LightCycler 480 system using SYBR-GREEN1 fluorescent reagents (Takara, Otsu, Shiga, Japan). Reactions were each carried out in 20 μL containing 0.4 μM (final concentration) of each primer (Table S3). The qPCR thermal profile consisted of 95 °C for 30 s, followed by 40 cycles of 95 °C for 10 s, 58 °C for 10 s, and 72 °C for 10 s. Dissociation curves were obtained from a thermal melting profile generated under a final PCR cycle of 95 °C for 5 s followed by a constant increase in temperature from 65 to 97 °C. Threshold values were empirically determined based on the observed linear amplification phase of all of the primer sets. Sample cycle threshold (*C*_t_) values were standardized for each template based on the reference gene control primer reaction, and the 2^−^^ΔΔ*C*t^ method was used to analyze relative changes in gene expression. Three biological replicates were used to ensure statistical credibility. SIGMASTAT from SPSS was used to analyze the qPCR data. Student’s *t* tests were used to detect differences between autotetraploid and diploid at the usual probability level *p* = 0.05.

## 5. Conclusions

In this study, phenotypic and transcriptomic changes between autotetraploid and diploid mulberry plants were compared. Larger plants and organs were observed in the autotetraploids. Only a few changes in gene transcriptional levels, including genes associated with plant hormones and photosynthesis, occurred in the autotetraploids. Further molecular mechanism-based studies of genome duplication events are still needed.

## Supplementary Materials

Supplementary materials can be found at http://www.mdpi.com/1422-0067/16/09/22938/s1.
