# Supplementary material for: Phenotypic and Transcriptomic Analyses of Autotetraploid and Diploid Mulberry (Morus alba L.)
Source: Int J Mol Sci. 2015 Sep 22;16(9):22938–56. doi: 10.3390/ijms160922938 (PMC4613344; doi:10.3390/ijms160922938)
Supplement: Supplementary file 1 [file ijms-16-22938-s001.zip › ijms-95456-Supplementary Information/Figures S1,S2 and Table S3.pdf]

Supplementary Information

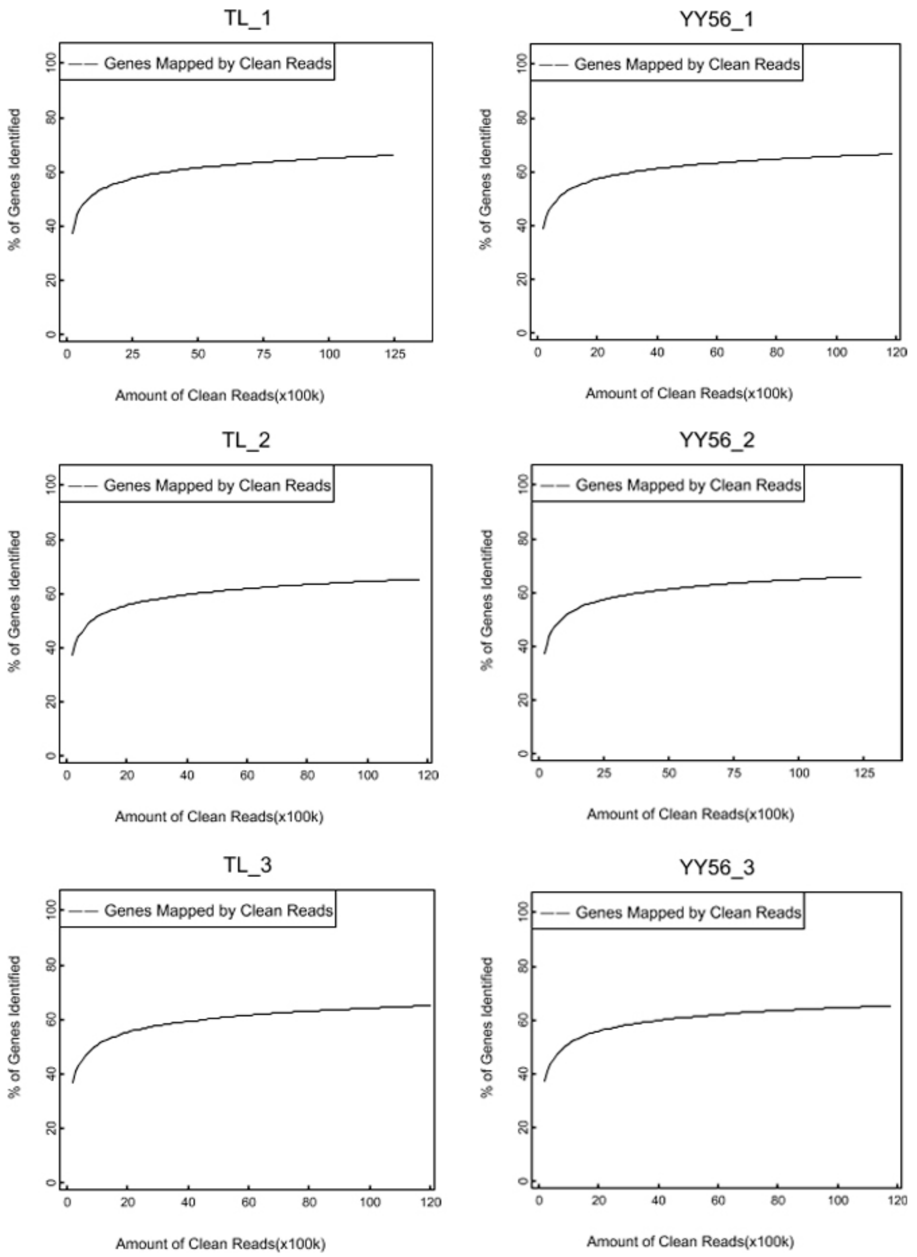

Figure S1. Sequencing saturation for all of the diploid (TL-1, -2, and -3) and autotetraploid (YY56-1, -2, and -3) samples. Each graph shows the genes mapped by clean reads.

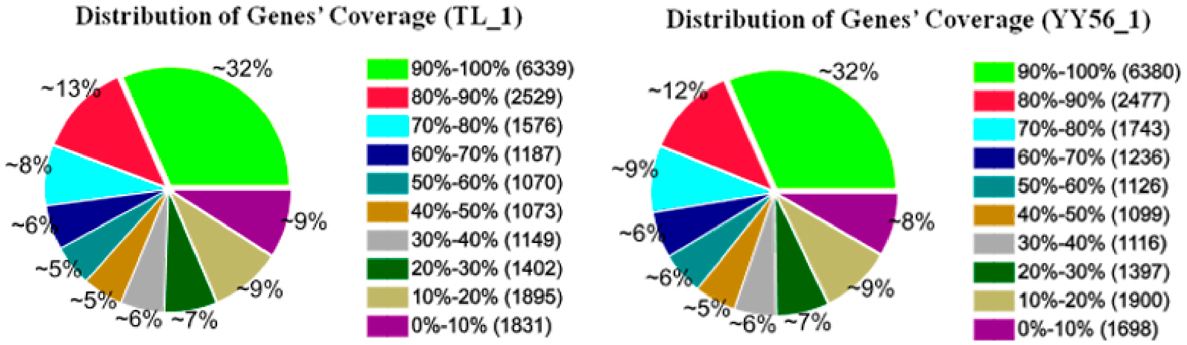

Figure S2. *Cont.*

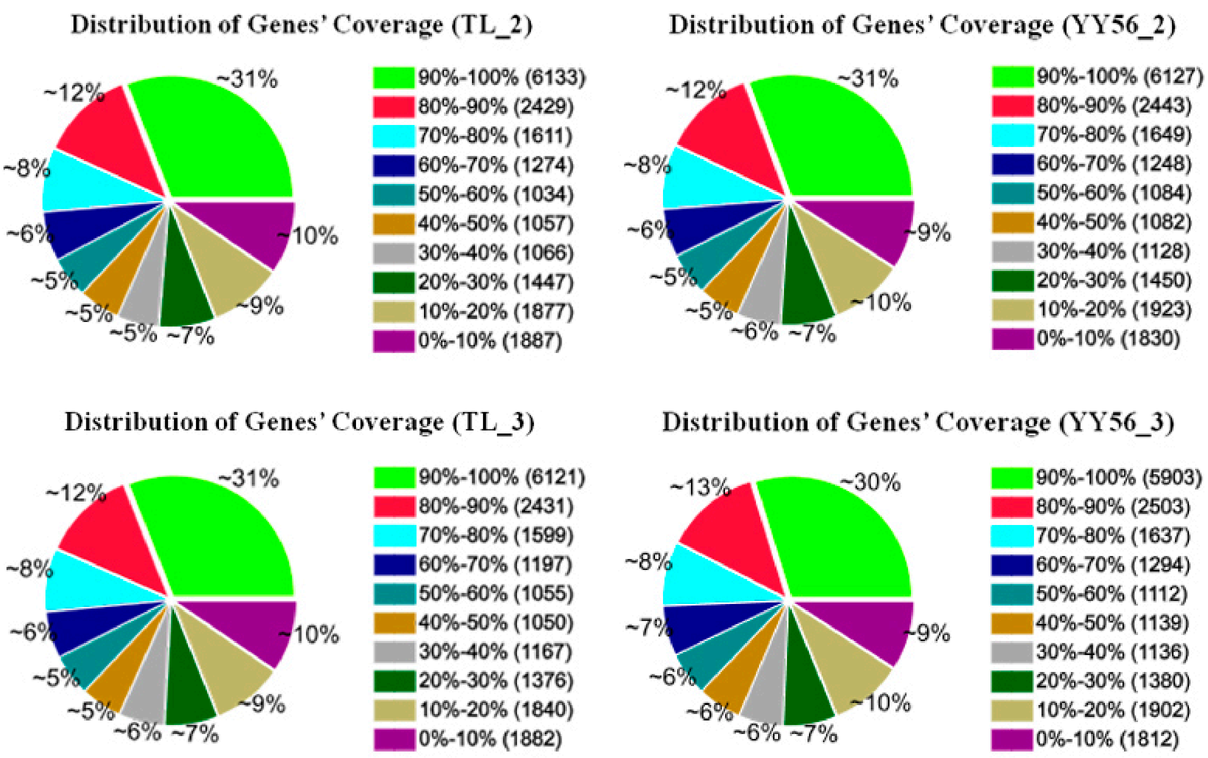

Figure S2. Distribution of gene coverage for the clean reads from all of the diploid (TL-1, -2, and -3) and autotetraploid (YY56-1, -2, and -3) samples.

Table S3. Primers used for qPCR.

Gene ID	Gene Annotation	Primer Sequence (5′–3′)
Morus007429	Photosystem I reaction center subunit XI	F: CAATCAACGGCGATCCCTTC<br>R: CCTGAGGAGTGGGCTGACTGC
Morus023580	Monothiol glutaredoxin	F: GGCGGTGTTTATAGGCGGCGA<br>R: TCAAACCCATAAAGCTCCAGCG
Morus013990	DELLA protein GAI	F: TTCCATACGGAAAGTTTGCTCACT<br>R: CAGTAGCCTCGATCATCTGAGACC
Morus010360	Phospholipase A1	F: AATGAGGCGTTTAGGAAGAGGGTA<br>R: GTAACGAACTTATGAGGGGCAAGCA
Morus017265	Chalcone isomerase	F: TTAGAGGTGGAGACACAGTGGTTTC<br>R: GACACGGCTTCATCCTTCAAGTACA
Morus010031	Cytokinin biosynthetic isopentenyltransferase	F: GGAAAGACAAGGTGGTTTTCGTGAT<br>R: ATTTTGTCGGAATTGATGATCTCG
Morus005139	Cytochrome C1	F: ATTCGTAGAGGTCACCAGGTCTACC<br>R: CTTTGTCTCTTCTTCAGCGTAAGCA
Morus012127	Polyphenol oxidase	F: CCTACCAACTGTTGCCCACCAATA<br>R: CAATGTACGCCTCGTCAACTGTATG
Morus013583	Calcium-binding protein	F: TGAAGATGGGAGGATGAGAAGAGAA<br>R: TCTCGTCAAACAGCGATGAAAGC
Morus011259	WRKY transcription factor	F: GTTCCCAATCATGGACTAAAATCAC<br>R: TGCGGGTGCATCTAAAGTAAGCC
